# The Hospital Officer's Bookshelf

**Published:** 1912-08-03

**Authors:** 


### THE HOSPITAL OFFICER'S BOOKSHELF.

Diseases of Women. By Florence E. Willey, M.D. (London: The Scientific Press. 1912. Pp. 92. Price 2s. net.)

This is a book designed for gynæcological nurses, being a reprint of certain lectures originally printed in *The Nursing Mirror.* The author's object has been to explain to the nurse the why and wherefore of the duties she is taught to carry out, so as to make her co-operation with the physician (or surgeon) more intelligent and more valuable. This does not mean that a nurse should acquire a text-book knowledge of gynæcology, but it does mean that she should have a general idea of the simple anatomy and physiology of the female pelvic organs, and of the morbid processes to which they are liable. The instruction given carries out this aim exceedingly well, neither erring on the score of undue technicality nor glossing over anything which nurses really ought to know and understand. There are a good many misprints—actually for actively, pulses for pubes, peritoneum for perineum, for instance.

Text-Book for Nurses of Anatomy, Physiology, Surgery, and Medicine. By E. W. H. Groves, F.R.C.S., and J. M. Fortescue-Brickdale, M.D. (Oxford University Press: Henry Frowde and Hodder and Stoughton. 1912. Pp. 407. Price 12s. 6d. net.)

The nurse who could master the contents of this comprehensive work, and assimilate them, would no doubt be a very fully instructed person. She would possess a vast quantity of knowledge, and knowledge is a thing of which no one can have too much. In fact, she would know a good deal more about anatomy and physiology than nine general practitioners out of ten do. And in surgery and medicine she would also be well up, though not perhaps quite so profoundly learned as an experienced clinician. At any rate, on paper she would possess a great deal of information—about every subject except nursing. Not that we would deprecate for a moment the instruction of nurses in the elements, the broad outlines, of all the four sciences of anatomy, physiology, surgery, and medicine. But to stuff a nurse's head full of the enormous mass of facts contained in this manual is to leave her scant time and only fuddled wits for the practical purpose of learning her profession. Admirable as an epitome of the subjects dealt with, the book is planned on too ambitious a scale; it is too encyclopædic for any but those who have had years of practical nursing experience and wish to delve rather deeply into medical science. Those actually undergoing training would not profit by reading the book, for it would only bemuse and muddle them. Nevertheless, it is only just to say that the work is admirably done in all its four sections; that it is fully up to date, and thoroughly reliable; that it is exceedingly well illustrated, and, as usual with the Oxford publications, excellently printed and produced.

The Treatment of Diseases of the Skin. By W. K. Sibley, M.D. (London: Edward Arnold. 1912. Pp. 280. Price 5s. net.)

We are not at all sure that the time is yet ripe for a monograph devoted solely to treatment in this branch of medicine. Neither the science of dermatology nor the general level of diagnostic skill among the profession at large has yet been so developed that it is safe to divorce diagnosis, etiology, and prognosis from methods of treating diseases of the skin. However, Dr. Sibley is evidently of another opinion. It is, in fact, avowedly with the object of directing attention to new, or comparatively new, methods that he has designed his book; and he starts out with fifty pages devoted to descriptions of such remedies as *x*-rays, electrolysis, ionisation, heat, cold, vaccines, and so forth. In these methods the author has a much greater faith than in drugs for many kinds of skin disease. Valuable as some of them are, when used with discretion for those diseases in which they have been definitely proved to be useful, the immediate resort to them for any and every case of skin disease is not only unnecessary but actually often inadvisable. Even from the purely commercial standpoint, they are often out of reach of the means of the patient. Dr. Sibley apparently recognises that some such criticism as this will be evoked by his book, for in the preface he describes himself as being in advance of the times. Perhaps by the time another edition of his book is required the times will have caught up with him; but we rather doubt it.

